# The Optimization and Characterization of an RNA-Cleaving Fluorogenic DNAzyme Probe for MDA-MB-231 Cell Detection

**DOI:** 10.3390/s17030650

**Published:** 2017-03-21

**Authors:** Pengcheng Xue, Shengnan He, Yu Mao, Long Qu, Feng Liu, Chunyan Tan, Yuyang Jiang, Ying Tan

**Affiliations:** 1 Department of Chemistry, Tsinghua University, Beijing 100084, China; xpc14@mails.tsinghua.edu.cn (P.X.); maoyu_gt@126.com (Y.M.); liu.feng@sz.tsinghua.edu.cn (F.L.); tancy@sz.tsinghua.edu.cn (C.T.); jiangyy@sz.tsinghua.edu.cn (Y.J.); 2 The Ministry-Province Jointly Constructed Base for State Key Lab-Shenzhen Key Laboratory of Chemical Biology, the Graduate School at Shenzhen, Tsinghua University, Shenzhen 518055, China; eheshengnan@163.com (S.H.); dradow@163.com (L.Q.)

**Keywords:** RFD probe, DNAzyme, optimization, MDA-MB-231 cell

## Abstract

Breast cancer is one of the most frequently diagnosed cancers in females worldwide and lacks specific biomarkers for early detection. In a previous study, we obtained a selective RNA-cleaving Fluorogenic DNAzyme (RFD) probe against MDA-MB-231 cells, typical breast cancer cells, through the systematic evolution of ligands by exponential process (SELEX). To improve the performance of this probe for actual application, we carried out a series of optimization experiments on the pH value of a reaction buffer, the type and concentration of cofactor ions, and sequence minimization. The length of the active domain of the probe reduced to 25 nt from 40 nt after optimization, which was synthesized more easily and economically. The detection limit of the optimized assay system was 2000 MDA-MB-231 cells in 30 min, which is more sensitive than the previous one (almost 5000 cells). The DNAzyme probe was also capable of distinguishing MDA-MB-231 cell specifically from 3 normal cells and 10 other tumor cells. This probe with high sensitivity, selectivity, and economic efficiency enhances the feasibility for further clinical application in breast cancer diagnosis. Herein, we developed an optimization system to produce a general strategy to establish an easy-to-use DNAzyme-based assay for other targets.

## 1. Introduction

Breast cancer, one of the most frequently diagnosed cancers, is the second leading cause of death by cancer in women [1,2]. The American Cancer Society (http://www.cancer.org) estimates that about 252,710 new cases of invasive breast cancer will be diagnosed in women, and about 40,610 women will die from breast cancer in the United States in 2017. In addition, the World Health Organization (WHO) reports an estimated 17 million deaths from breast cancer by 2030 [3]. Therefore, breast cancer remains a tremendous threat to the human health system. Clinical studies have demonstrated that the survival rate of patients is closely related to the time of disease diagnosis [4].

Early detection and timely therapy of breast cancer has long been considered to be a vital strategy in the control of the disease’s development, which can lead to timely care of patients and prevent potential tumor metastasis [5,6]. Considering the importance of early detection, many conventional techniques, such as touch detection, imaging methods, biopsy, mammography, and serum tumor markers detection, have been established for breast cancer detection [6]. However, these detection techniques suffer from false-positive/negative results and are complicated, time-consuming, expensive, and lack efficiency [7,8,9]. Therefore, there is a great need to develop effective molecular probes that are highly specific, sensitive, and convenient for the early diagnosis of breast cancer. The development of new techniques to identify and characterize tumor cells unambiguously, simply, and rapidly is of great importance [10,11]. In recent years, functional nucleic acids—such as aptamers and DNAzymes—as effective molecular tools have attracted increasing attention [12,13]. For instance, Tan’s group used the cell-SELEX method to generate molecular probes for specific recognition of cancer cells, including human Burkitt’s lymphoma cells [14], liver cancer cells [15], and acute leukemia cells [16]. Aptamers possess many advantages, such as a low molecular weight, easy modification, reproducible synthesis, fast tissue penetration, low toxicity or immunogenicity, easy storage, and high binding affinity and selectivity [17,18,19,20,21,22]. DNAzymes, or DNA enzymes [23], are synthetic, single-stranded DNA molecules with the ability to catalyze various chemical reactions, which have been generated using in vitro selection. DNAzymes have also become a prevalent tool in a broad range of interesting applications, including biosensing and gene therapeutics, based on their attractive properties of good chemical stability and ease of synthesis [24].

The number and popularity of RNA-cleaving DNAzymes from in vitro selection using random sequencing libraries has increased, of which 10–23 [25] and 8–17 [25] are the best known RNA-cleaving DNAzymes. Li and colleagues developed a bio-sensing platform named ‘RNA-cleavage fluorogenic DNAzyme’ (RFD) which can cleave a substrate composed of a lone RNA linkage and two deoxyribonucleotides labeled with a dye/quencher pair [25,26,27,28,29]. Furthermore, the RFD probe can realize a ‘mix-and-read’ [26,30] system of assays. Compared to conventional approaches, the RFD system is also more time-efficient and convenient. Many detection systems with high sensitivity and selectivity based on a RFD probe have been reported in recent years, including detection of *Escherichia coli* (*E. coli*) [26,31], *Clostridium difficile*, [32], MDA-MB-231 cells [29], T47D cells [33], etc. Although these special RFDs offer an excellent opportunity for designing biosensor-based DNAzymes, only a few studies optimized the RFD probes after in vitro selection [27,34,35,36,37]. Some shortcomings presented by the original probes obtained from the selection include high cost and less sensitivity, which extremely limit the application of the probes. Thus, further research is quite necessary to optimize the RFD probes to recognize and detect targets more efficiently. In a previous work, we obtained a selective RFD probe (AAI2-5) that is capable of distinguishing MDA-MB-231 cells (a model breast cancer cell line) from normal cells and other types of tumor cells. AAI2-5 was the first RFD probe used to detect cancer cells and tumor samples, which showed good characteristics for recognizing MDA-MB-231 cells. From a theoretical perspective, RFD offers a new platform for the detection of breast cancer. In this work, we performed a series of experiments to optimize and study the characterization of the RFD probe based on the pH value of the reaction buffer, the type and concentration of cofactor ions, and sequence minimization. The optimization promotes the development of a general strategy to establish an easy-to-use and DNAzyme-based assay for other targets.

## 2. Experimental Section

### 2.1. Materials and Reagents

All the T4 DNA ligase, T4 polynucleotide kinase (PNK) and ATP were purchased from New England Biolabs Ltd. (NEB, Beijing, China). Ampicillin, N,N,N′,N′-tetramethylenediamine (TEMED), and a 40% solution of acrylamide/bis-acrylamide (29:1) mixture was purchased from Thermo Fisher Scientific (Shanghai, China). The cytoplasmic/nuclear protein extraction kits and BCA protein assay kits were obtained from Tiangen Biotech Co. Ltd. (Beijing, China). L-15 medium was purchased from Gibco (Carlsbad, CA, USA). Dulbecco’s modified Eagle’s medium (DMEM) and Iscove’s modified Dubecco’s medium (IMDM) were obtained from Hyclone (Logan, UT, USA). All other chemicals were purchased from Sigma-Aldrich (St. Louis, MO, USA). All the involved chemicals were of analytical reagent grade and used without further purification. The water used in this study was double deionized (ddH_2_O) and autoclaved with a Milli-Q Synthesis from a Millipore system. All DNA oligonucleotides were synthesized by Takara Biotechnology Co. Ltd. (Dalian, China). All DNA oligonucleotides were purified by 10% denatured PAGE before use except that the FAM-labeled DNA was purified by HPLC. The selection buffer (2×) contained 400 mM NaCl, 100 mM HEPES (pH 7.5), and 10 mM MgCl_2_. Lysis buffer contained 10 mM HEPES (pH 7.9), 10 mM KCl, 1 mM EDTA, 0.1% NP40, and 1× complete Protease Inhibitor Cocktail. All solutions were freshly prepared before use.

### 2.2. Cell Lines and Cell Culture

All cell lines used in this study were purchased from the cell bank, Shanghai Institutes for Biological Sciences. The MDA-MB-231 cell line (mammary gland adenocarcinoma) was cultured in L-15 medium supplemented with 10% FBS in air 100%. The MCF 10A (mammary gland fibrocystic disease), ZR-75-1 (mammary gland ductal carcinoma), BT-474 (mammary gland ductal carcinoma), SK-BR-3 (mammary gland adenocarcinoma), K562 (chronic myelogenous leukemia), NCI-H69 (small cell lung cancer), CCRF-CEM (acute lymphoblastic leukemia), QSG-7701 (normal liver), and Hela (cervical adenocarcinoma) cell lines were cultured in RPMI-1640 medium supplemented with 10% FBS in a humidified atmosphere of 5% CO_2_ and 95% air. The MCF-7 (mammary gland adenocarcinoma), HEB (normal brain), and U251 (glioblastoma) cell lines were cultures in DMEM supplemented with 10% FBS. The HCT 116 (colorectal carcinoma) cell line was cultured in IMDM supplemented with 10% FBS in a humidified atmosphere of 5% CO_2_ and 95% air. All cells were cultured at 37 °C. 

### 2.3. Sample Preparation

All kinds of cells were washed twice with 1× PBS and scraped into cell lysis buffer. The cell resuspensions were sonicated for 4 s and suspended for 9 s, this operation was repeated three times. The suspensions were incubated on ice for 30 min, while being inverted every 5 min. Finally, the cell lysate was centrifuged for 15 min (20,000 g, 4 °C). The supernatant was collected and stored in −80 °C for further use.

### 2.4. Sequences Ligation Process

Standard and modified DNA oligonucleotides were synthesized by Takara Biotechnology Co. Ltd. (Dalian, China). The ligation reaction was performed as follows and the DNA sequences used in the ligation process are summarized in Table 1.

Substrate (100 pmol) was first phosphorylated with a small amount of ATP (100 nmol) and PNK (0.2 U/μL, final concentration, the total reaction volume is 100 μL) for 30 min at 37 °C. Then the mixture was heated to 90 °C for 5 min in order to inactivate the PNK. Next, this mixture was directly added into a solution containing 120 pmol AL (the sequence obtained through SELEX and not ligated with substrate) and 120 pmol DNA template. The whole system was heated at 90 °C for 50 s to completely inactivate the PNK and despiralize the DNA sequence and cooled to room temperature. The ligation was initiated by the addition of T4 DNA ligase (0.2 U/μL, final concentration and final volume was 200 μL) and continued at room temperature for 90 min. The ligated DNA was subsequently purified by 10% denatured polyacrylamide gel electrophoresis (dPAGE), followed by ethanol precipitation (repeated three times). The purpose of the operation was to separate the ligated DNA from unligated DNA. It should be noted that the substrate contained a fluorescein-labeled thymine deoxyribonucleotide (Fluorescein-dT, F), a adenine ribonucleotide (R) as the cleavage site and a DABCYL-labeled thymine deoxyribonucleotide (DABCYL-dT, Q). 

## 3. Results and Discussion

**Scheme 1:**
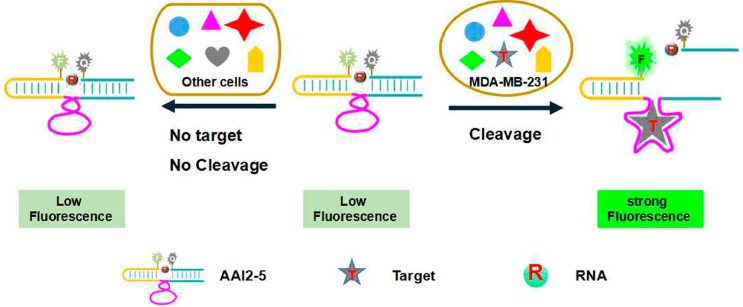
The working principle of the probe.

According to a previous study [29], the AAI2-5 probe was modified with fluorescein and DABCYL to show green fluorescence excitation at 485 nm and to induce fluorescence quenching, respectively. Under natural conditions, fluorescein-dT became closer to DABCYL-dT, leading to quenched fluorescence. When the target was introduced, the catalytic ability of the probe was activated, followed by the cleaving of the substrate, increasing the distance between DABCYL-dT and fluorescein-dT and increasing the fluorescence signal (Scheme 1). It was found that the cleavage of the substrates could generate a nearly six-fold increase in fluorescence in the PAGE-based experiment [27,29,34,38,39,40,41]. We calculated the percentage of cleavage using the formula [34]

Clv% = (F_Int[clv]_/6)/(F_Int[clv]_/6 + F_Int[unclv]_) × 100%

where F_Int[clv]_ is the intensity of the cleaved DNA band and F_Int[unclv]_ represents the intensity of the uncleaved DNA band.

### 3.1. Optimization of the Reaction Buffer Conditions

**Figure 1:**
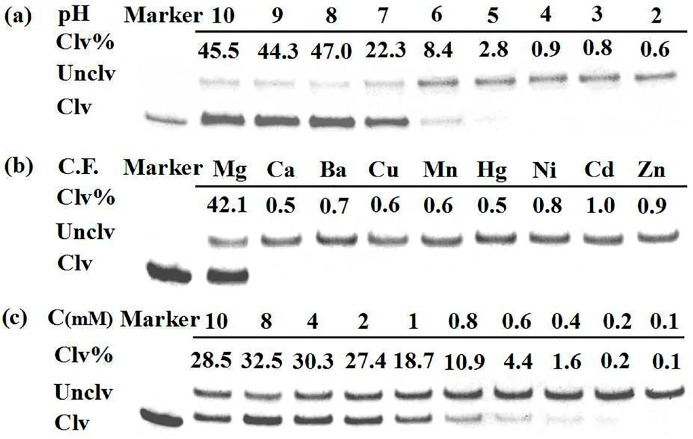
Optimization of the reaction buffer. 10% dPAGE analysis of probe incubated with MDA-MB-231 cell lysate for 60 min. (**a**) The cleavage efficiency of AAI2-5 with different pH environments; (**b**) The cleavage efficiency of AAI2-5 with different divalent ions in reaction buffer; (**c**) The cleavage efficiency of different Mg^2+^ concentrations. Clv% = cleavage efficiency; Unclv = uncleaved probe; Clv = cleaved probe; C.F = cofactor types; C (mM) = cofactor concentration

The reaction condition was mainly affected by the pH value of the reaction buffer as well as the type and concentration of cofactor ions. We optimized these conditions one by one. For all cleavage reactions conducted in this part, the reaction system contains 1× selection buffer, 0.5 pmol/μL RFD probe, 0.2 mM EGTA, and 30 μg/mL cell lysate. The mixture was incubated for 60 min at room temperature. The cleavage product was separated by 10% dPAGE. As shown in Figure 1a, the catalytic activity only occurred in the pH range from 7 to 10, which is an alkaline condition. Because more OH^−^ are present in alkaline conditions, the inherent transesterification reaction may occur more easily between a phosphodiester linkage and the nearby 2-hydroxyl group [28]. Therefore, the catalytic ability of the DNAzyme was stronger under the selected alkaline conditions [42]. However, we did not test a reaction in pH greater than 10 considering that the probe was cleaved in higher pH conditions without the target [28]. According to the data, the maximum catalytic rate occurred at pH 8, which was similar to the bio-system and was chosen as the best pH condition in subsequent experiments. 

In the RFD catalytic reaction, bivalent ions play an important and necessary role as metal cofactors [43,44,45,46,47,48,49]. In this work, we studied eight other common bivalent ions (Ba^2+^, Ca^2+^, Cu^2+^, Fe^2+^, Ni^2+^, Zn^2+^, Mn^2+^, Hg^2+^) as replacements of Mg^2+^ (10 mM) in the selection buffer. Generally speaking, Mg^2+^ (or another divalent metal cation) as a cofactor can accelerate the cleavage of a particular RNA phosphoester [23]. The divalent metal ions bind to specific sites and form a stable and active structure. Interestingly, in our experiments, all eight metal cations scarcely stimulated the enhancement of fluorescence signal (Figure 1b). A possible explanation is that Mg^2+^ has unique physical and chemical properties, such as metal coordination number, ionic radius, electrophilicity, oxidation state, and natural affinity with RNA [42,43]. Therefore, we selected Mg^2+^ as the best cofactor for subsequent experiments.

We then studied the effect of the concentration of Mg^2+^ on the activity of DNAzyme. We incubated different concentrations of Mg^2+^, from 0.1 mM to 10 mM, in the reaction system for 60 min at room temperature. The results are shown in Figure 1c. Considering that the rate of cleavage is largely dependent on the concentration of Mg^2+^, the cleavage efficiency was enhanced when the concentration of Mg^2+^ increased and a stable cleaving value appeared at a concentration of 4 mM. The probe requires Mg^2+^ as a cofactor to catalyze the RNA; thus, more Mg^2+^ could accelerate the cleavage of the particular RNA phosphoester. Interestingly, we found that 4 mM is also the saturated concentration of Mg^2+^ in the reaction, meaning that a higher concentration is not necessary. A lower concentration of the cofactor reduced the ionic effect in the nonspecific interaction, which is more applicable for practical implementation of the probe. 

### 3.2. Sequence Minimization

In the original selection experiment, we established a library of 40-nt single-stranded DNA molecules, which acted as the active domain to recognize the target and cleave the substrate. The main reason for using a relatively long DNA sequence was that a longer DNA sequence may result in better catalytic efficiency [50,51,52]. Although DNA with long sequences have been isolated, the crucial domain pertains to only a shorter piece of the sequence, such as 8–17 [25] and 10–23 [25]. Therefore, we explored the active domain of the RFD probe, AAI2-5 (A0), for its ability to recognize MDA-MB-231 cells with relatively limited nucleotides via sequence minimization.

**Figure 2:**
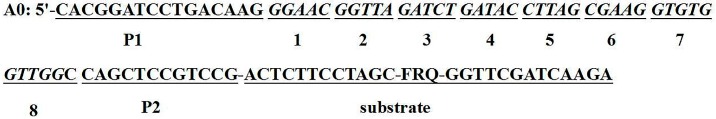
The original sequence of AAI2-5 (A0) which was divided into four parts including primer sequences P1 and P2, substrate, and active domain (italic letters) which was further divided into eight units.

The obtained sequence of A0 in the previous work is shown in Figure 2. For the convenience of description, we divided the original A0 into four parts including P1, P2, catalytic domain and substrate. The parts of P1 and P2 were used as primers to amplify sequences in the selection process. The sequence signed in italic was catalytic domain which binds with target and cleaves the substrate. And the sequence modified with F, R, Q was substrate which could generate the fluorescence signal. Then, the catalytic domain was further divided into eight units with 5-nt each. In this strategy, the catalytic ability of the probes was scored by the cleavage efficiency after the reaction: a cleavage percentage less than 10% with a minus sign (−); 10%–20% with one plus sign (+); 20%–30% with two plus signs (++); and cleavage activity greater than 30% indicated that the deleted sequence had little effect on the probe activity and was represented by three plus signs (+++). All truncation sequences were examined using A0 as the reference deoxyribozyme.

**Figure 3:**
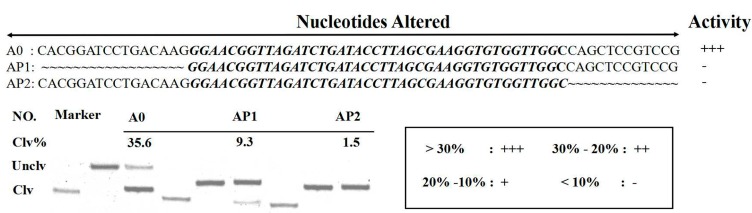
The cleavage efficiency of the original sequence (A0) and two sequences without primers (AP1 and AP2). 10% dPAGE analysis of each probe incubated with MDA-MB-231 cell lysate for 60 min. A0 = AAI2-5; Clv% = cleavage efficiency; Unclv = uncleaved probe; Clv = cleaved probe.

**Figure 4:**
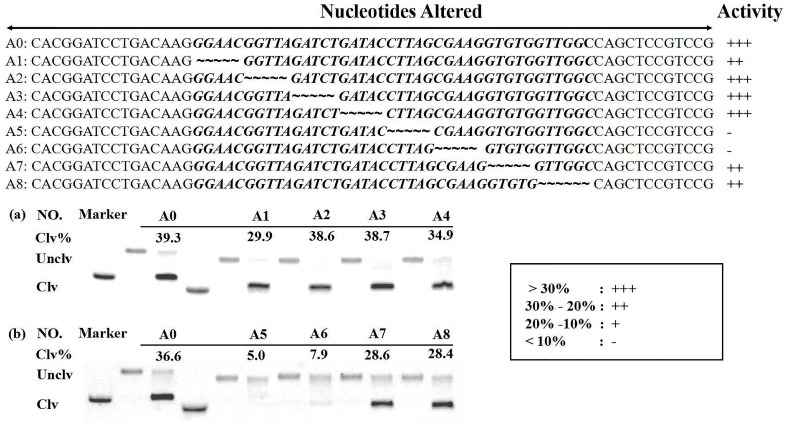
The truncated sequences A1–A8 and the activity of each sequence. 10% dPAGE analysis of each probe incubated with MDA-MB-231 cell lysate for 60 min. Clv% = cleavage efficiency; Unclv = uncleaved probe; Clv = cleaved probe.

In the truncation design, the substrate domain remained constant, which ensured that the signal would be detected and other parts would be truncated to varying degrees. To first explore the function of the primes in the catalytic reaction, we truncated the P1 part-generating AP1 sequence and P2 part-generating AP2 sequence, respectively as:

Figure 3 shows that only the original sequence, A0, exhibited a good cleavage percentage, while AP1 and AP2 presented apparent activity reduction with cleavage percent less than 10%. These results indicated that the two primer sequences are essential for maintaining the conformation of this RFD probe. In the next experiments, we reserved these two parts and then optimized the active domain using nucleotide truncation. We sequentially removed any 5-nt unit from the 5′ to 3′ end of the active domain and named the sequences A1–A8 (Figure 4).

**Figure 5:**
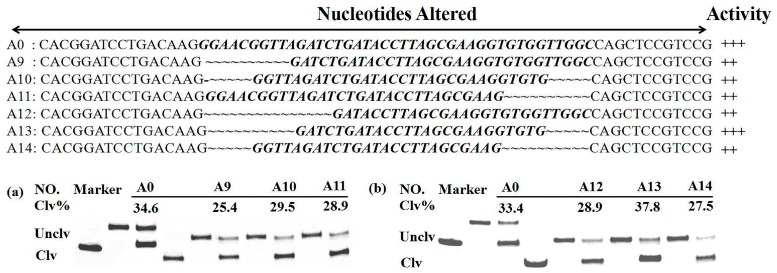
The truncated sequences A9–A14 and the activity of each sequence. 10% dPAGE analysis of each probe incubated with MDA-MB-231 cell lysate for 60 min. Clv% = cleavage efficiency; Unclv = uncleaved probe; Clv = cleaved probe.

Interestingly, we found that all probes could cleave the substrate efficiently except for deletion unit 5 (A5, -) and unit 6 (A6, -) sequences. A5 and A6 presented poor catalytic activity, meaning that these 10 nucleotides are required for the catalytic function of deoxyribozyme, may engage in secondary interactions, and are responsible for substrate recognition. Therefore, we reserved these two units in the next minimization experiments. After the first sequence deletion round, we designed further truncation sequences with 10-nt and 15-nt deletion with constant units 5 and 6. Another six sequences named A9–A14 were obtained, which were found to exhibit medium (A9, A10, A11, A12, and A14) or good activity (A13). And the results were shown in Figure 5. It is important to mention that the A13 sequence showed a higher cleavage activity than A0.

These results suggest that unit 1, unit 2, and unit 8 were dispensable in the catalytic reaction, while units 3–7 played an important role in maintaining the active structure. In this work, the active domain of the probe was reduced to 25 nt from 40 nt after optimization, which was synthesized more easily and economically.

### 3.3. Sensitivity of the Optimized Probe

**Figure 6:**
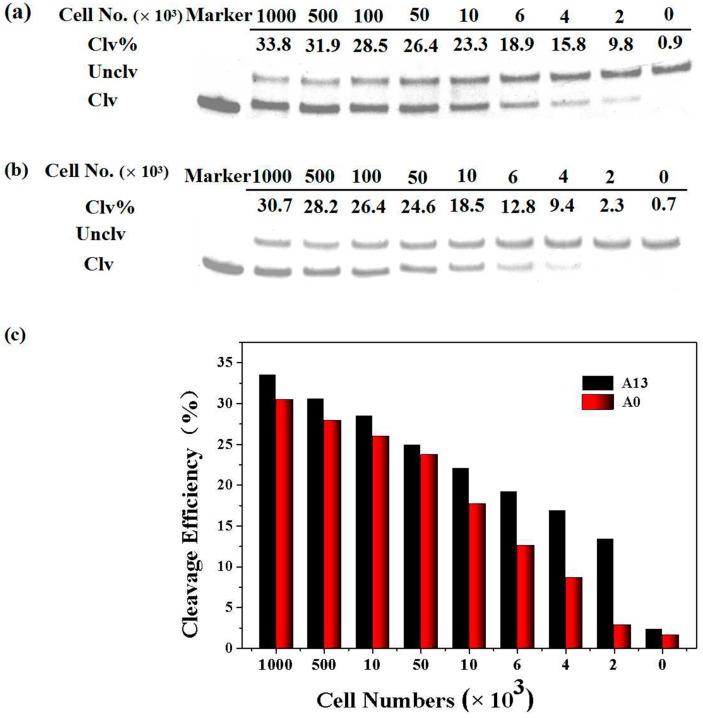
The sensitivity of two probes. 10% dPAGE analysis of each probe incubated with MDA-MB-231 cell lysate for 60 min. (**a**) A13 and (**b**) A0 were incubated with different MDA-MB-231 cell numbers; (**c**) A bar graph of the two probes' detection limit. Clv% = cleavage efficiency; Unclv = uncleaved probe; Clv = cleaved probe.

According to the results, the original probe, A0, detected 0.5 μg/mL (nearly 5000 cell/mL) cell lysate proteins of MDA-MB-231 cells [29]. In this work, we tested the detection limit of A0 and A13 (Figure 6). 0.5 pmol/μL probe A0 and A13 were incubated with 1× selection buffer, 0.2 mM EGTA, and different concentrations of MDA-MB-231 cell lysate, separately. To describe more conveniently, we used cell numbers to represent the concentration of the target. A0 showed 12.8% and 9.4% cleavage when the cell concentration was 6000 cell/mL and 4000 cell/mL, respectively. Compared to A0, A13 with 9.8% cleavage presented a better sensitivity when the cell concentration was 2000 cell/mL. In other words, through a series of optimization experiments, we obtained a more sensitive RFD probe for MDA-MB-231 cells. Probe A13 with a shorter sequence may fold a simpler, more preferable, and effective structure, while deleting some dispensable bases can also reduce the steric hindrance effect. The results suggest that our optimization enhanced the sensitivity of the assay and the feasibility of the probe’s application.

### 3.4. Specificity of the New Probe

**Figure 7:**
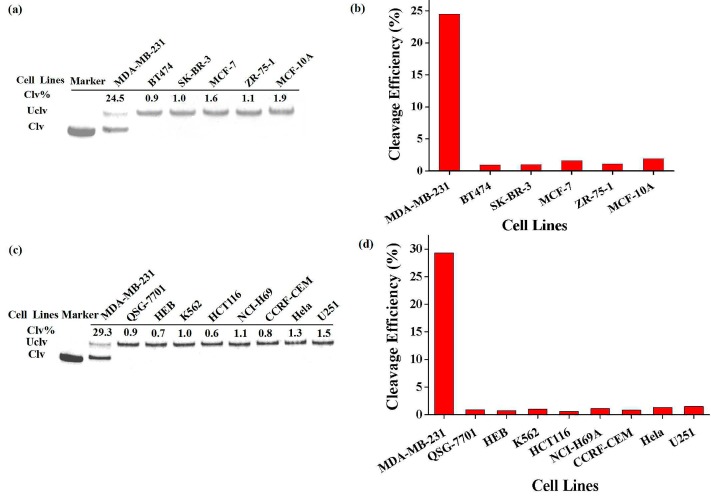
The specificity of A13. (**a**,**b**) A13 was incubated with 60 μg/mL of different breast cell line lysates for 30 min. MCF-10A are normal breast cells, while others are breast cancer cells. (**c**,**d**) A13 was incubated with 60 μg/mL of different cell line lysate for 30 min. QSG 7701 is normal human liver cell line, HEB is normal human brain cell line, and other cells are cancer cell lines. Clv% = cleavage efficiency; Unclv = uncleaved probe; Clv = cleaved probe.

To investigate the specificity of the optimized probe, we subsequently tested the response of A13 to various cell lysates produced by different cell lines, including 3 normal cell lines (QSG 7701, HEB, MCF-10A) and 10 tumor cell lines (BT474, SK-BR-3, MCF-7, ZR-75-1, K562, HCT116, NCI-H69, CCRF-CEM, Hela, and U251), which contain breast cancer, liver cancer, small cell lung cancer, etc. All cell lysates were quantified using a BCA protein assay kit. In this experiment, 0.5 pmol/μL probe A13 was incubated with 60 μg/mL MDA-MB-231 cell lysate, 1× selection buffer, and 0.2 mM EGTA for 30 min and displayed significant cleavage efficiency, as shown in Figure 7. This probe is capable of specifically distinguishing MDA-MB-231 cells from normal cells and other tumor cells. These results strongly prove that the optimized probe A13 has excellent specificity like A0.

## 4. Conclusions

In this work, we successfully optimized and characterized the RFD probe for MDA-MB-231 cells through series experiments, including reaction condition optimization and sequence minimization. We reduced the cofactor concentration of Mg^2+^ to 4 mM, which weakened the ionic effect in the nonspecific interaction and is useful for practical application of the probe. After sequence truncation, we obtained a better probe, A13, which is 16-nt shorter than the initial probe, A0. Probe A13 presented excellent specificity and could distinguish MDA-MB-231 cells from 10 other tumor cell lines and 3 normal human cell lines. More importantly, A13 could detect as low as 2000 MDA-MB-231 tumor cells, which is more sensitive than probe A0. In summary, the shorter length of the probe displayed good sensitivity, excellent specificity, and more economical efficiency. These properties enhance the feasibility of the probe for further clinical application in breast cancer diagnosis. Based on the results, our optimization system may be efficiently used to develop a general strategy to establish an easy-to-use DNAzyme-based assay for other targets.

## Figures and Tables

**Table 1:** DNA sequence used in the experiment.

Name	Sequences 5′-3′
Substrate	5′-ACTCTTCCTAGC-FRQ-GGTTCGATCAAGA-3′
AL	5′-CACGGATCCTGACAAGGGAACGGTTAGATCTGATACCTTAGCGAAGGTGTGGTTGGCCAGCTCCGTCCG-3′
Template 1	5′-CTAGGAAGAGTCGGACGGAGCTG-3′
Template 2	5′-CTAGGAAGAGTGCCAACCACACC-3′
